# Effects of oropharyngeal administration of own mother's milk on oral microbial colonization in very low birth weight infants fed by gastric tube: A randomized controlled trial

**DOI:** 10.1002/iid3.1247

**Published:** 2024-04-17

**Authors:** Jie Liu, Xiyang Zhang, Qian Zhao, Xiaohe Mu, Chuanzhong Yang, Yan Ning, Xiaoyun Xiong, Xiaoling Qin, Lilian Chen

**Affiliations:** ^1^ Department of Traditional Chinese Medicine Gynecology, Pediatric Neurorehabilitation Department, Department of Neonatology Shenzhen Maternity and Child Healthcare Hospital Shenzhen China; ^2^ School of Nursing Shanxi University of Chinese Medicine Taiyuan Shanxi China; ^3^ Department of Critical care medicine Shaanxi Province Kangfu Hospital Xi'an China

**Keywords:** human milk, microbiota, oropharynx, very low birth weight infants

## Abstract

**Aims:**

The aim of the present study was to explore the effect of oropharyngeal mother's milk administration on oral microbial colonization in infants fed by gastric tube at different time points.

**Methods:**

Infants (*n* = 116) with birth weight <1500 g were randomly allocated into two groups which both received breast milk for enteral nutrition. The control group (*n* = 51) accepted oropharyngeal normal saline administration. The experimental group (*n* = 53) accepted oropharyngeal mother's milk administration before fed by gastric tube once every 3 h over 21 days after birth. We analyzed the oral microbiota at initiation and 7 and 14 and 21 days later using 16S DNA amplicon sequencing.

**Results:**

There were no difference in oral microbial diversity between the two groups at any time point, but diversity decreased significantly over time in both groups. On the first day of life, the oral microbiota of the infant in the experimental and control groups consisted mainly of Firmicutes (7.75%, 6.18%) and Proteobacteria (68.65%, 68.69%), respectively. As time increases to 21 days after birth, Firmicutes (77.67%, 77.66%) had replaced Proteobacteria (68.65%, 68.69%) as the predominant phylum.

**Discussion:**

From birth to 21 days after birth, oropharyngeal mother's milk administration did not change the diversity and structural composition of the oral microbiota. The oral microbial diversity of infants declined significantly over time. Firmicutes had replaced Proteobacteria as the predominant phylum.

## Graphical abstract

The oral microbial diversity of infants fed by gastric tube declined significantly over time. Firmicutes had replaced Proteobacteria as the predominant phylum at day of life 21.

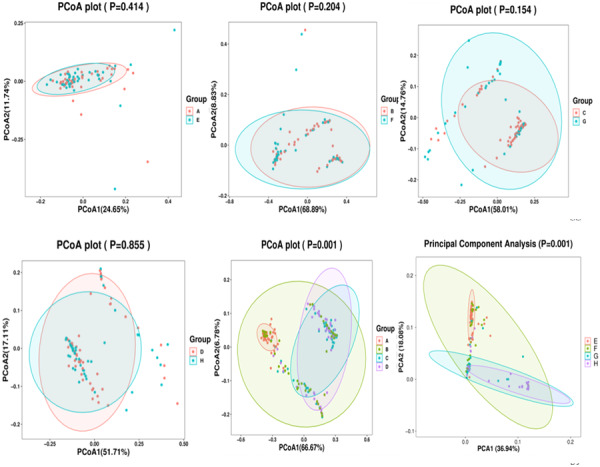

## 1 INTRODUCTION

Although medical care has improved, there are still significant morbidity and mortality rates for very low birth weight infants. Low birth weight has been associated with increased risks of neonatal mortality.^1^, ^2^ Premature infants are at greater risk of death in low and medium income countries.^3^ They have immature systemic development and low immunity, which makes them susceptible to various infections and affects the success rate of treatment. Long‐term complications and prolonged hospitalization can result in substantial costs to families, hospitals, and society. For infants weighing 751–1000 g, a single bloodstream infection could extend hospitalization by 7 days and increase hospital costs by $12,480.^4^ Therefore, improving the survival rate of very low birth weight infants and reducing the incidence of infection are still significant challenges.

The oral microbial community, consisting of over 760 species of bacteria, is one of the richest microbiota in the human body.^5^ Changes in the composition and structure of oral microbial community play an important role in the development and progression of disease. Normally, They maintain a dynamic balance with the body and are among the non‐specific immune factors in the body.^6^, ^7^, ^8^ Once there is a persistent imbalance in the microbial community, it may affect the body's digestive metabolism and immune function, and is closely related to the development of diseases such as neonatal necrotizing enterocolitis (NEC) and ventilator‐associated pneumonia. At present, A number of cross‐sectional studies have focused on the intestinal microbial community and there has been less research about the oral microbial community, especially on the colonization and evolution of the oral microbial community in tube‐fed very low birth weight infants.

Very low birth weight infants do not receive breastfeeding directly from the mother's breast for a short time at birth and require tube feeding for several days to weeks after birth. Because coordination between suckling, swallowing and breathing are not developed until 32–34 weeks of gestational age.^9^ The lack of exposure of the oral mucosa to breast milk in tube fed infants hinders the transmission of microflora between mother and infant, which may lead to differences in oral microbial community between preterm infants and healthy newborns for a period of time after birth. At the same time, invasive operations such as indwelling gastric tube and ventilator‐assisted breathing may lead to the propagation of a large number of pathogens in the mouth, which is easy to cause local or systemic infection.^10^ Researchers have proposed that a small amount of breast milk can be directly in contact with the oropharyngeal mucosa of tube‐fed preterm infants through syringes or sterile swabs. This intervention is also known as oral immune therapy.^11^ Components in breast milk provide barrier protection to prevent pathogenic bacteria from adhering to the oropharyngeal mucosa.^12^ This may reduce the incidence of NEC and ventilator‐associated pneumonia in preterm infants.^13^ Other study found that the dominant family of oral microbiota in the study group at 48 and 96 h after oropharyngeal administration of mother's milk was Planococcaceae, while in the control group the dominant family were Moraxellaceae and Staphylococcaceae, respectively, but there was no significant difference in the incidence of infections between the two groups of infants. This may be related to the relatively short duration of the intervention.^14^ The mechanism of oropharyngeal breast milk administration needs to be further investigated.

The purpose of this study was to investigate the effect of oropharyngeal mother's milk administration on oral microbiota in preterm infants fed by gastric tube, and to analyse the changes in the species and diversity of the oral microbiota in preterm infants during the first 21 days of life.

## 2 MATERIALS AND METHODS

### 2.1 Ethics

The study protocol was approved by the Ethics Committee of the Shenzhen Maternity & Child Healthcare Hospital (SFYLS[2020]038). In addition, the clinical trial was registered at the Chinese Clinical Trial Registry with number ChiCTR2100046645 (24/5/2021). Parental informed consent was obtained before study enrollment for each infant. Informed consent was obtained from parents within 2 h of birth.

### 2.2 Subjects

Premature infants who meet the following inclusion criteria were enrolled in the study: birth weight ≤1500 g, transferred to Neonatal Intensive Care Unit (NICU) within 2 h after birth, fed by gastric tube, parents agree to provide breast milk.

Exclusion criteria were: infants with congenital oral and gastrointestinal anomalies; maternal history of positive human immunodeficiency virus (HIV) status or syphilis; the mother being in the acute stage of infection that prevents her from providing breast milk, including severe mastitis, fungal infections of the breast or nipple that require treatment.

The shedding criteria were as follows: infant was requested to withdraw at any stage of the trial by parents; infant was transferred to other hospitals or died before the end of the trial; mothers can not provide colostrum for their infants in the first 96 h of life.

### 2.3 Collection of breast milk

Breast milk collection was completed by the maternal and her family members. On the day the infants were admitted to hospital, parents were taught about breastfeeding (such as how is breast milk extracted, stored and transported to the hospital) by nurses.

Parents wash their hands thoroughly with hand sanitizer before breastfeeding and touching breast pump, a process that lasts at least 15 s, paying attention to cleanliness around the fingernails, and drying them with disposable paper towels or wipes. Parents wash and high temperature boil sterilized breast pumps after use. Once received the breast milk, the nurse checked the infant's information against the label on the storage bottle and used 1 mL sterile syringe to draw up 0.3 mL volume (According to the summary of the preliminary trial that 0.3 mL milk can just soak a medical sterile swab). Nurses carried sterile gloves during operation, strictly according to the aseptic technique. The syringes stored at 2–6°C in a specified milk refrigerator, they were available for 24 h. Breast milk used for intestinal feeding was refrigerated or frozen depending on the feeding situation of preterm infants in accordance with our NICU feeding protocol.

### 2.4 Study design

This randomized controlled clinical trial was conducted from August 2020 to April 2022 in the NICU of the Shenzhen Maternity and Child Healthcare Hospital, China. All infants (*n* = 116) were randomized into a experimental group or a control group from 1:1 ratio random number table generated by computer. Of the 116 preterm infants, 12 dropped out of the study due to death or lack of breast milk. The control group routinely accepted oropharyngeal administration of normal saline every shift. On this basis, The oropharyngeal administration of mother's milk was started immediately after obtaining breast milk and was given every 3 h for the 21 days in the study group. This procedure performed 5 min before each gavage feeding. The buccal swab served to evenly distribute the milk over the cheeks, gums, tongue surface and sublingual. Swabs were applied to the oral cavity quickly (<5 s per side) and gently. Nurses carried sterile gloves during operation, strictly according to the aseptic technique. The session was discontinued and recorded if any of the following issues developed: bradycardia (hazard ratio [HR] < 100/min) or tachycardia (HR > 200/min), respiratory rate (RR > 80/min), apnea and pulse oxygen saturation (SpO2 < 90%). After the end of the intervention, the infants were continued to be observed for more than 30 s. The physician established the infant's feeding program and probiotic usage. Both groups received breast milk for enteral nutrition with orogastric tube. Formula feeding only when breast milk was insufficient. Parenteral nutrition was not discontinued until infants were receiving an enteral feeding volume equal to 100 mL/kg/day.

### 2.5 Demographic and outcome data

Maternal demographics, containing age, prenatal diseases and medication status. Infant demographic information was collected and compared. The first part of the record included gender, gestational age at birth, birth weight, number of babies, Apgar score, delivery mode, etc. The second part of the data document registered the type and dosage of tube‐fed milk for premature infants within 21 days, as well as the time and frequency of oropharyngeal administration of mother's milk.

### 2.6 Specimen collection and assays

Specimens were collected on the day of birth (within 2 h of birth), on the 7th, 14th, and 21st days after birth. The investigator wore sterile gloves during collection and used a sterile swab to gently wipe the inner cheek surface of each infant. The swabs were placed in 1.5 mL sterile tubes and stored immediately in a refrigerator at −80°C.

DNA extractions DNA from different samples was extracted according to manufacturer's instructions. Nuclear‐free water was used for blank. The total DNA was eluted in 50 μL of Elution buffer and stored at −80°C until measurement in the PCR. The V3‐V4 region of the prokaryotic (bacterial and archaeal) small‐subunit (16S) rRNA gene was amplified with primers 341F (5′‐CCTACGGGNGGCWGCAG‐3′) and 805R (5′‐GACTACHVGGGTATCTAATCC‐3′). The 5′ ends of the primers were tagged with specific barcods per sample and sequencing universal primers. PCR amplification was performed in a total volume of 25 μL reaction mixture containing 25 ng of template DNA, 12.5 μL PCR Premix, 2.5 μL of each primer, and PCR‐grade water to adjust the volume. The PCR conditions to amplify the prokaryotic 16S fragments consisted of an initial denaturation at 98°C for 30 s; 32 cycles of denaturation at 98°C for 10 s, annealing at 54°C for 30 s, and extension at 72°C for 45 s; and then final extension at 72°C for 10 min. The PCR products were confirmed with 2% agarose gel electrophoresis. Throughout the DNA extraction process, ultrapure water, instead of a sample solution, was used to exclude the possibility of false‐positive PCR results as a negative control. The PCR products were purified by AMPure XT beads (Beckman Coulter Genomics) and quantified by Qubit (Invitrogen). The amplicon pools were prepared for sequencing and the size and quantity of the amplicon library were assessed on Agilent 2100 Bioanalyzer (Agilent) and with the Library Quantification Kit for Illumina (Kapa Biosciences), respectively. The libraries were sequenced on NovaSeq PE250 platform.

Samples were sequenced on an Illumina NovaSeq platform according to the manufacturer's recommendations, provided by LC‐Bio. Paired‐end reads was assigned to samples based on their unique barcode and truncated by cutting off the barcode and primer sequence. Paired‐end reads were merged using FLASH. Quality filtering on the raw reads were performed under specific filtering conditions to obtain the high‐quality clean tags according to the trim (v0.94). Chimeric sequences were filtered using Vsearch software (v2.3.4). After replication using DADA2, we obtained feature table and feature sequence. Alpha diversity and beta diversity were calculated by QIIME2, which the same number of sequences were extracted randomly through reducing the number of sequences to the minimum of some samples, and the relative abundance (X bacteria count/total count) is used in bacteria taxonomy. Alpha diversity and Beta diversity were analyzed by QIIME2 process, and pictures were drawn by R (v3.5.2). The sequence alignment of species annotation was performed by Blast, and the alignment database was SILVA and NT‐16S.

### 2.7 Statistics

SPSS 26.0 was used for data analysis. The Student *t*‐test was used to compare continuous parametric variables, Mann–Whitney *U*‐test was used for continuous nonparametric variables, and the Chi‐square test or the Fisher exact test was used for categorical variables when appropriate. Shannon index and Simpson index were calculated as a measure of genus diversity. Principal coordinates were computed for the weighted distance matrices and used to generate principal coordinate analysis (PCoA) plots. Linear discriminate analysis effect size (commonly referred to as LEfSe) utilizes the Kruskal–Wallis test and the Wilcoxon test on subclasses to determine a signed (positive or negative) log score to estimate a biological effect. A *p* value of <.05 was considered statistically significant.

## 3 RESULTS

**Figure 1:**
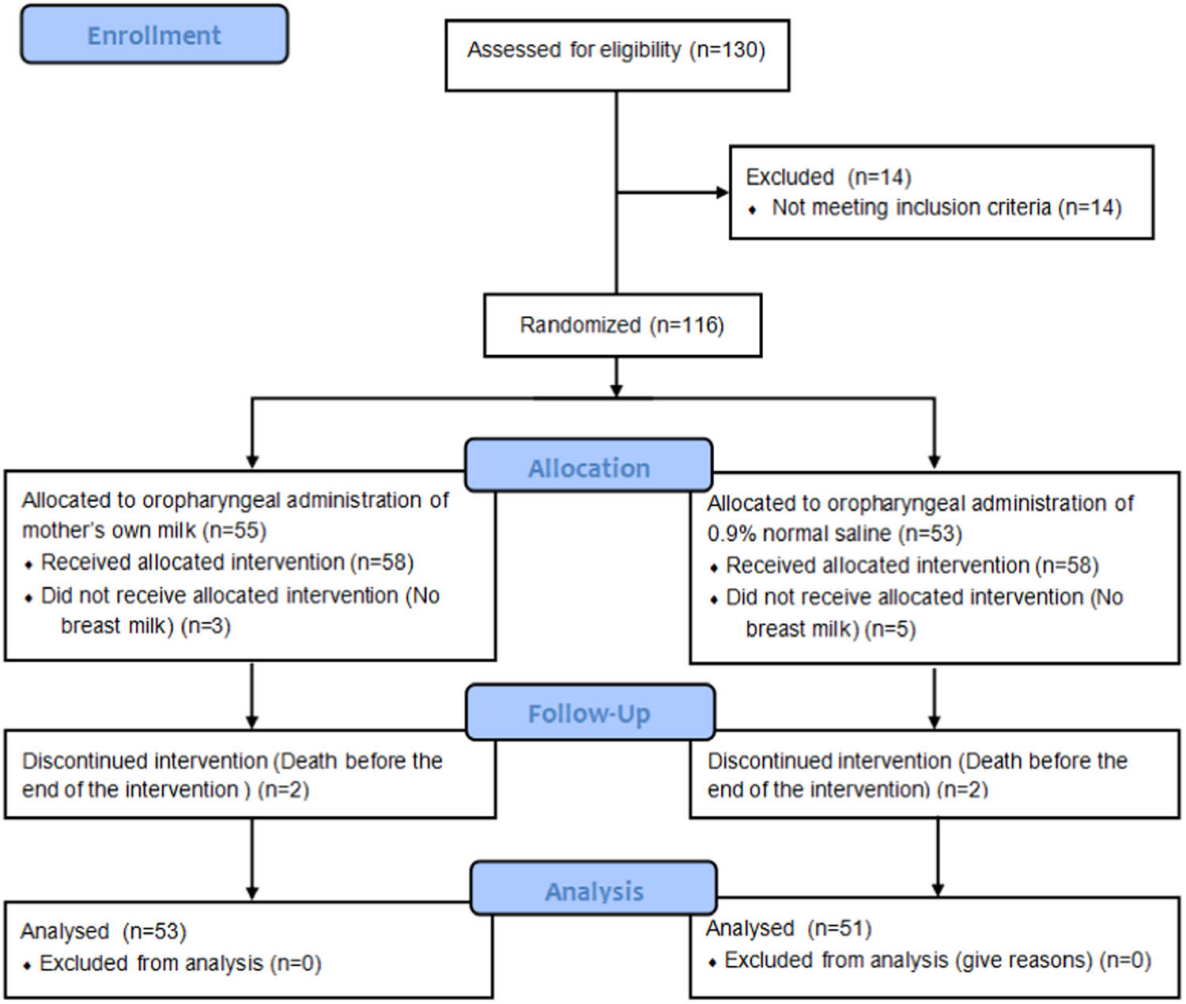
CONSORT diagram.

A total of 116 subjects were enrolled in the study. Among the participants, five infants in the experimental group and seven infants in the control group dropped out because they had no milk availability after 96 h postpartum or died before the end of the intervention (Figure 1). No significant (*p* > .05) differences were found in the baseline characteristics between both groups (Table 1).

**Table 1:** Maternal and infant characteristics.

Infant characteristics	Study group	Control group	*t/χ* ^2^
Male *n* (%)	27 (50.9)	28 (54.9)	0.163
Gestational age at birth (wk, M ± *SD*)^a^	29.356 ± 2.000	29.420 ± 2.62	−0.141
Birth weight (kg, M ± *SD*)	1.205 ± 0.256	1.114 ± 0.242	1.848
Apgar score at 1 min median (1st, 3rd quartile)	9 (8, 10)	9 (8, 10)	0.555
Apgar score at 5 min median (1st, 3rd quartile) (quartiles)	10 (10, 10)	10 (10, 10)	0.922
Vaginal delivery *n* (%)	21 (39.6)	18 (35.3)	0.208
Invasive mechanical ventilation *n* (%)	6 (11.3)	10 (19.6)	1.371
Resuscitation *n* (%)	5 (9.4)	4 (7.8)	0.083
Feeding for 3 weeks after birth *n* (%)			0.366
Breast milk	26 (49.1)	22 (43.1)	
Mixed	27 (50.9)	29 (56.9)	
Antibiotics *n* (%)	33 (62.3)	34 (66.7)	0.220
Days of antibiotics (M ± *SD*)	5.830 ± 2.953	5.862 ± 3.212	−0.054
Probiotics *n* (%)	31 (58.5)	32 (62.7)	0.197
Started oral feeding on day 7 *n* (%)	0 (0)	0 (0)	NA
Started oral feeding on day 14 *n* (%)	3 (5.7)	4 (7.8)	0.197
Started oral feeding on day 21 *n* (%)	8 (15.1)	10 (19.6)	0.370
**Maternal characteristics**			
Gestational diabetes *n* (%)	12 (22.6)	15 (29.4)	0.620
Gestational hypertension *n* (%)	5 (9.4)	9 (17.6)	1.505
Premature rupture of membranes *n* (%)	27 (50.9)	21 (41.2)	0.998
Clinical chorioamnionitis *n* (%)	0 (0)	0 (0)	NA
Antenatal steroid *n* (%)	45 (84.9)	45 (88.2)	0.247
Antenatal antibiotics *n* (%)	20 (37.7)	13 (25.5)	1.799

Abbreviations: M ± SD, means ± standard deviation; *n* (%), number (%); *t*, independent samples t‐test; χ^2^, Chi‐square test; NA, not applicable.

**Figure 2:**
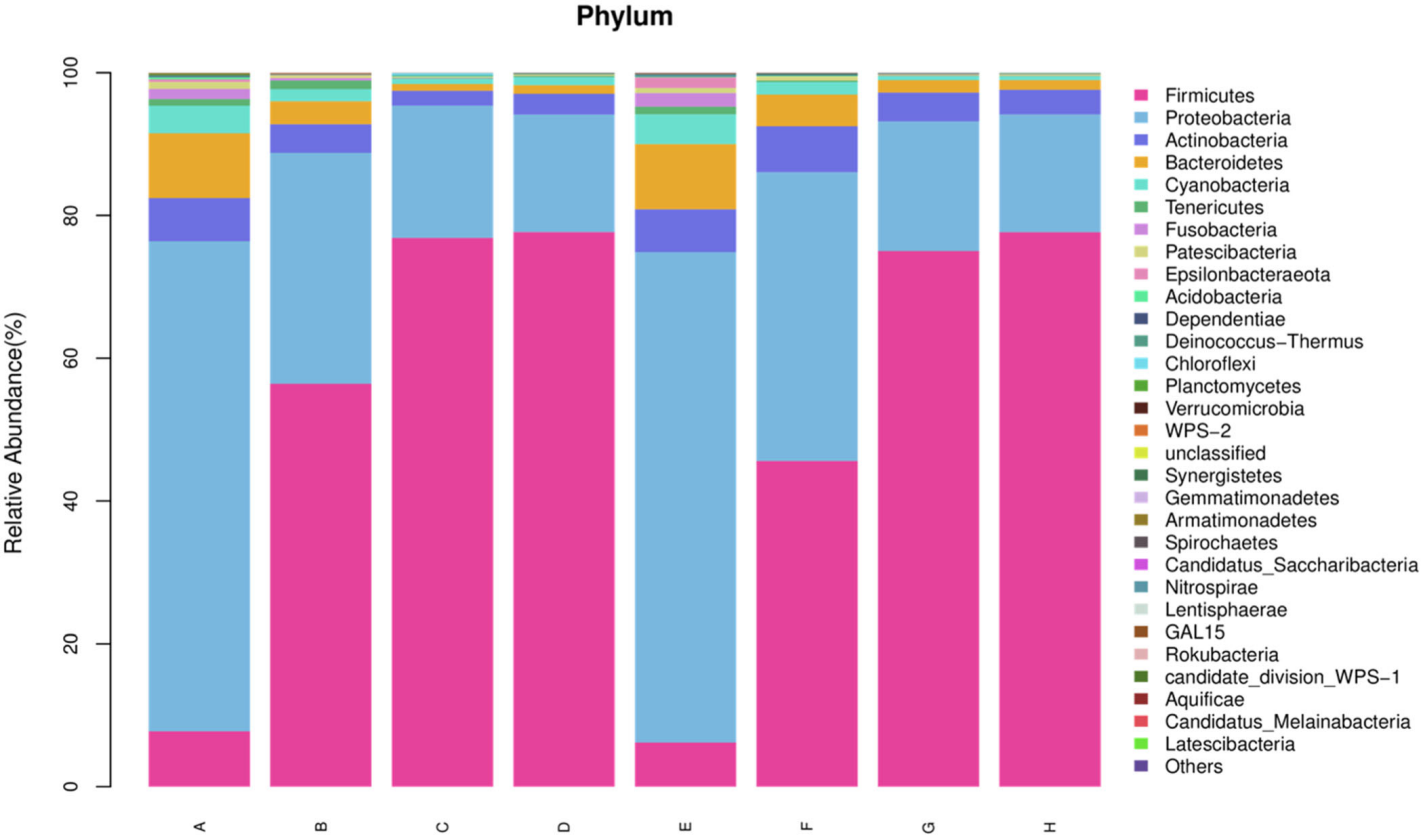
Mean relative abundance of oral bacteria at the phylum level for each group at birth, 7th, 14th, and 21st days. A: Birth in the experimental group, B: 7th day of postnatal life in the experimental group, C: 14th day of postnatal life in the experimental group, D: 21st day of postnatal in the experimental group. E: Birth in the control group, F: 7th day of postnatal life in the control group, G: 14th day of postnatal life in the control group, H: 21st day of postnatal in the control group.

Analysis of relative abundance of species showed that the oral flora was dominated by Firmicutes, Proteobacteria, Actinobacteria, Bacteroidetes which account for more than 90% (Figure 2). The proportion of Firmicutes in the study and control groups was 7.75% and 6.18% on the first day of life, respectively, and then gradually increased to reach a peak of 77.67% and 77.66% at the 21st postnatal day. The proportion of Proteobacteria in the experimental and control groups on the first postnatal day was 68.65% and 68.69%, respectively, and then gradually decreased to 16.48% and 16.51% at the 21st postnatal day. The proportions of Actinobacteria and Bacteroidetes were downtrending with time (Table 2).

**Table 2:** Proportions of Firmicutes, Proteobacteria, Actinobacteria, and Bacteroidetes (%).

Phylum	A	B	C	D	E	F	G	H
Firmicutes	7.75	56.41	76.86	77.67	6.18	45.60	75.01	77.66
Proteobacteria	68.65	32.36	18.53	16.48	68.69	40.45	18.18	16.51
Actinobacteria	6.05	4.01	2.09	2.91	6.00	6.45	4.05	3.45
Bacteroidetes	9.09	3.21	0.95	1.20	9.13	4.44	1.72	1.35

*Note*: A: Birth in the experimental group, B: 7th day of postnatal life in the experimental group, C: 14th day of postnatal life in the experimental group, D: 21st day of postnatal in the experimental group. E: Birth in the control group, F: 7th day of postnatal life in the control group, G: 14th day of postnatal life in the control group, H: 21st day of postnatal in the control group.

**Figure 3:**
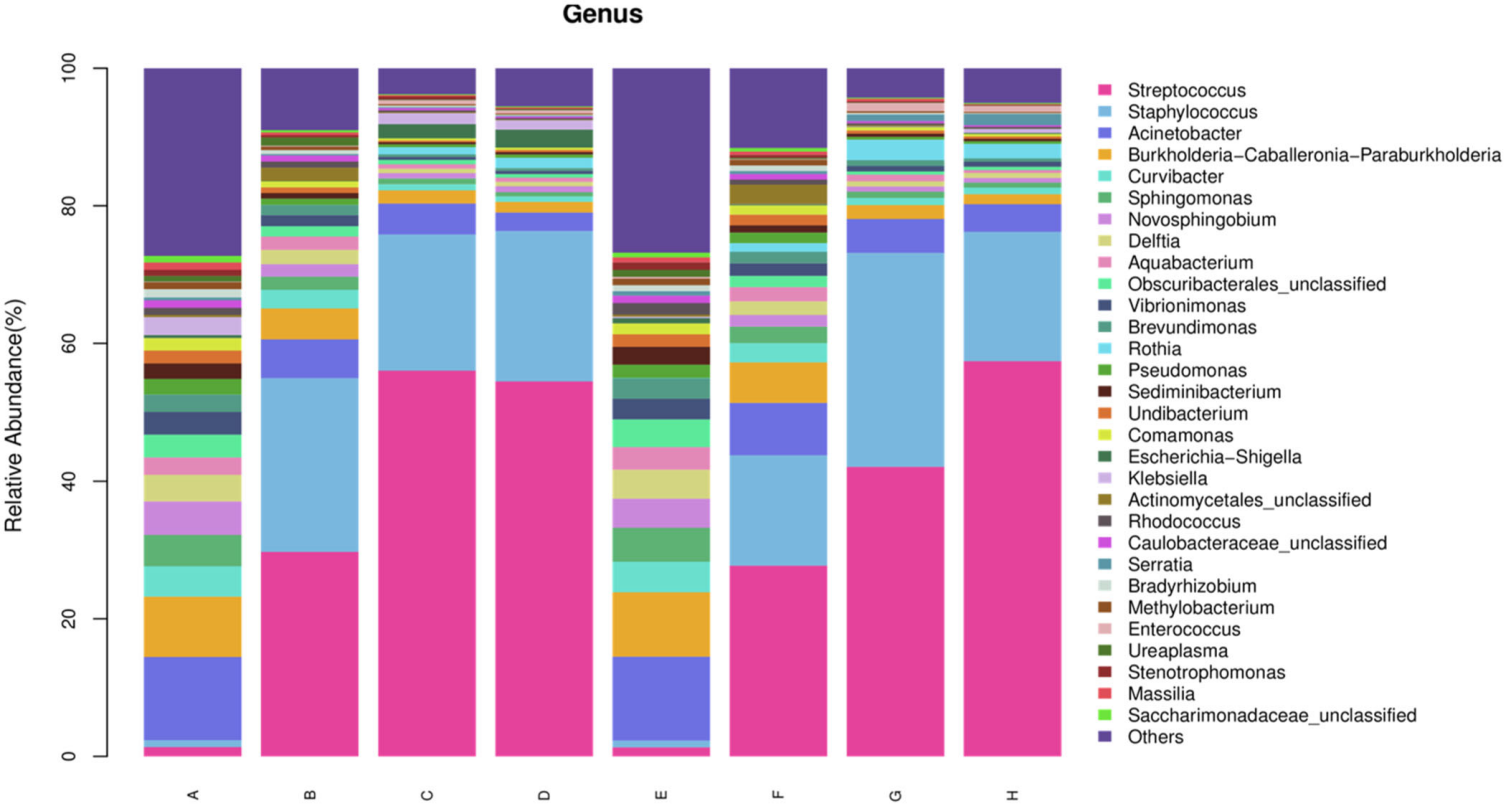
Mean relative abundance of oral bacteria at the genus level for each group at birth, 7th, 14th, and 21st days. A: Birth in the experimental group, B: 7th day of postnatal life in the experimental group, C: 14th day of postnatal life in the experimental group, D: 21st day of postnatal in the experimental group. E: Birth in the control group, F: 7th day of postnatal life in the control group, G: 14th day of postnatal life in the control group, H: 21st day of postnatal in the control group.

Analysis of relative abundance of species showed that the oral flora at the genus level was dominated by Streptococcus, Staphylococcus, Acinetobacter, and Burkholderia‐Caballeronia‐Paraburkholderia (Figure 3). The proportion of Streptococcus in the study and control groups was 1.35% and 1.32% on the first day of life, respectively, and then gradually increased to reach a peak of 54.47% and 57.41% at the 21st postnatal day. The proportion of Staphylococcus in the experimental on the first postnatal day was 0.99% and then gradually decreased to 21.87% at the 21st postnatal day. The proportion of Staphylococcus in the control group was 31.05% at the 14th postnatal day and then declined to 18.81% at the 21st postnatal day. The proportions of Acinetobacter and Burkholderia‐Caballeronia‐Paraburkholderia were downtrend with time (Table 3).

Figure 4Comparison of the alpha diversity was evaluated using Violin Plot. Shannon diversity index and Simpson diversity index were used to estimate the alpha diversity of the oral microbiota. A: Birth in the experimental group, B: 7th day of postnatal life in the experimental group, C: 14th day of postnatal life in the experimental group, D: 21st day of postnatal in the experimental group. E: Birth in the control group, F: 7th day of postnatal life in the control group, G: 14th day of postnatal life in the control group, H: 21st day of postnatal in the control group.

**Figure:**
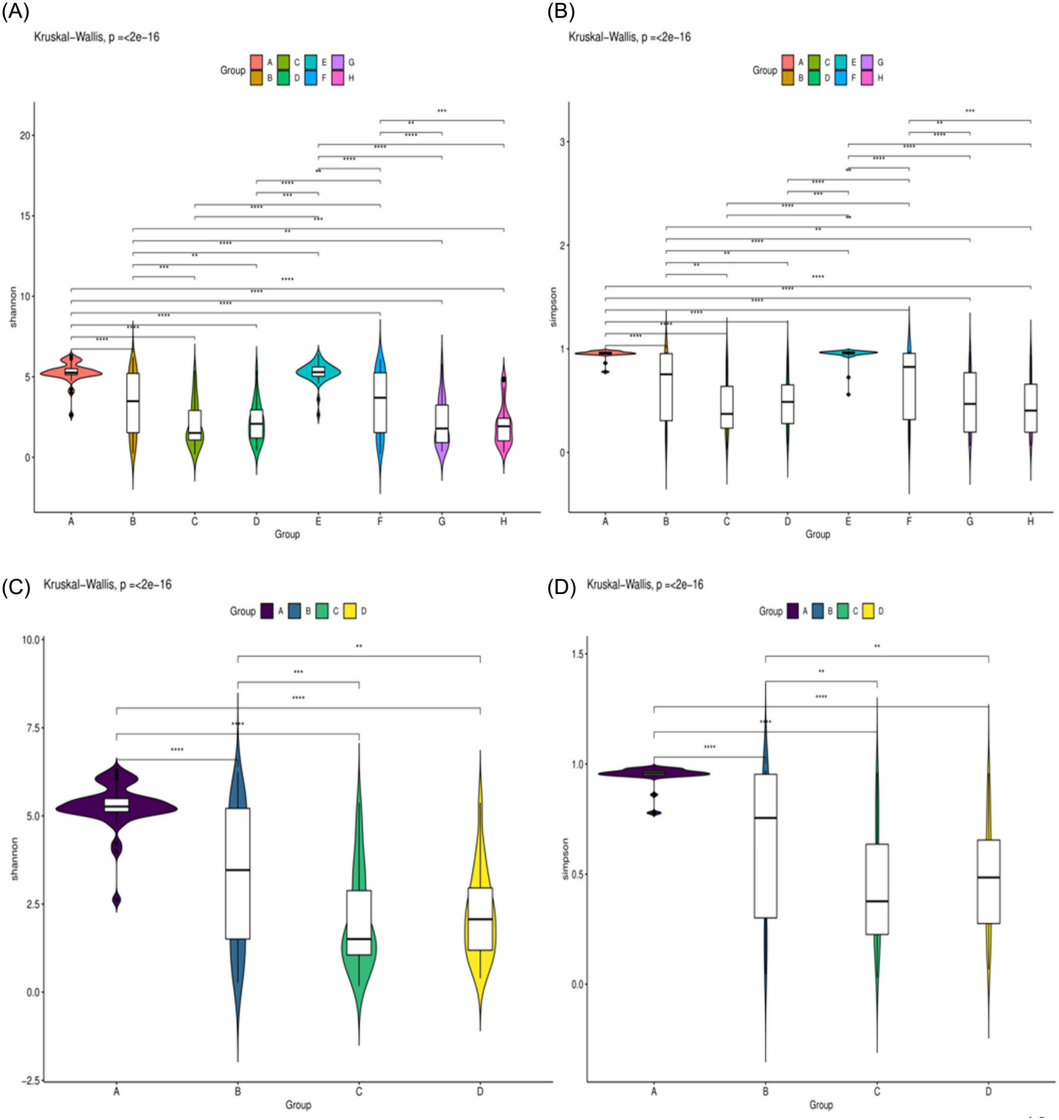

**Figure:**
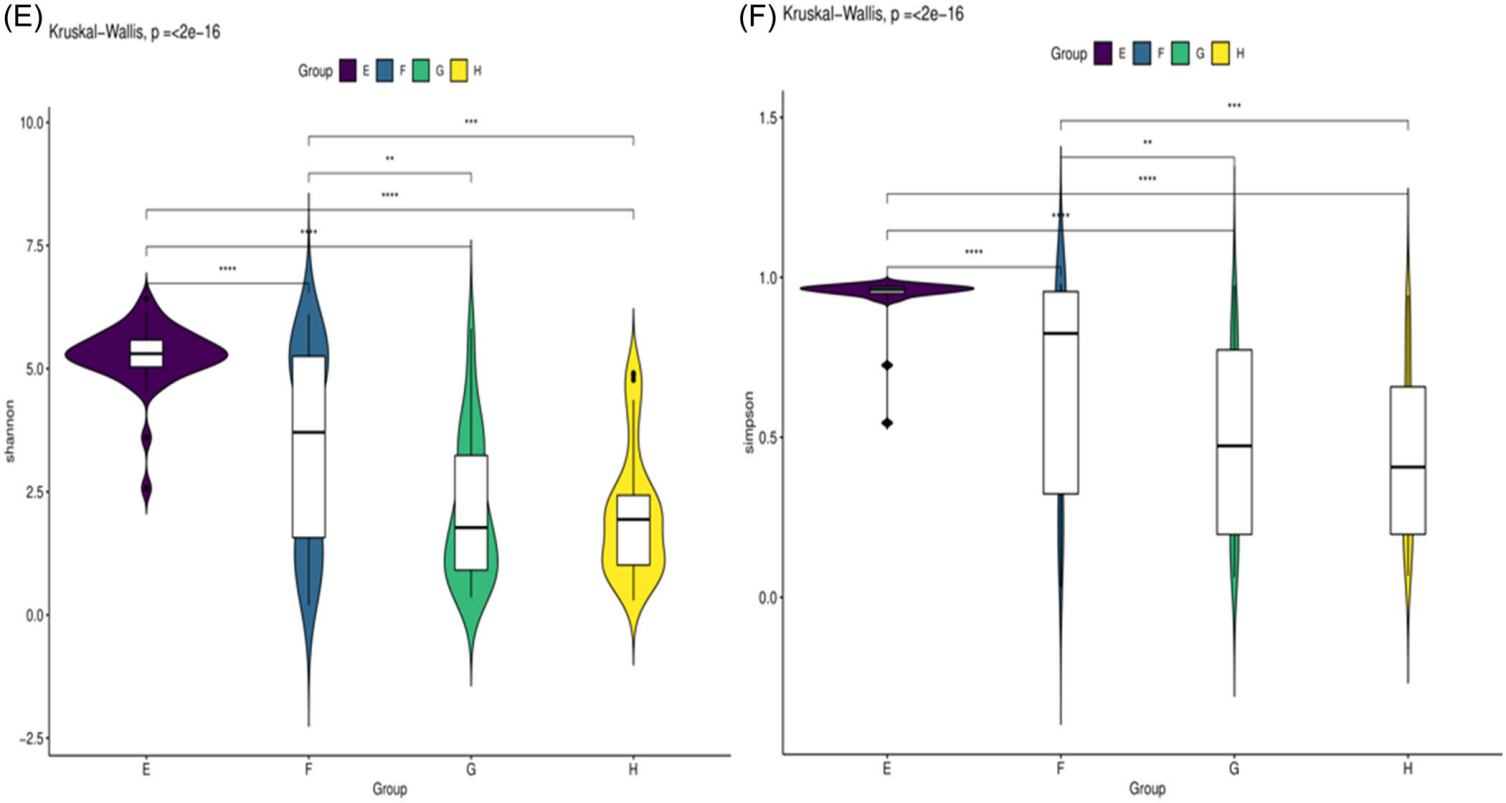

α‐Diversity as measured by Shannon index and Simpson index was unchanged between study and control groups on the 1st, 7th, 14th, and 28th days after birth (*p* > .05). However, the α‐diversity of the oral flora was highest on the first day of life and significantly dropped over time in infants across both groups (*p* < .05) (Figure 4).

**Figure 5:**
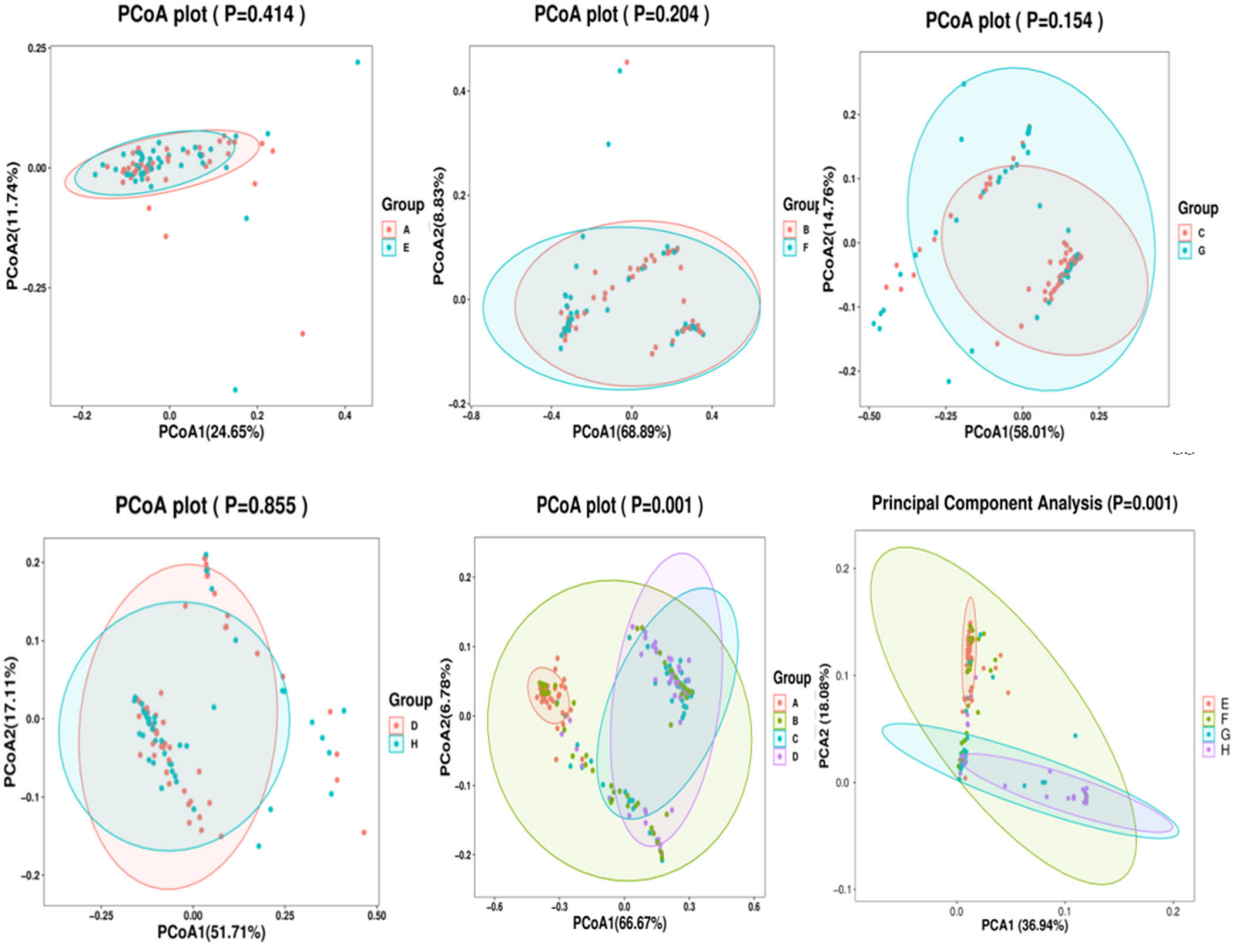
PCoA of bacterial beta diversity based on the weighted UniFrac distances. A: Birth in the experimental group, B: 7th day of postnatal life in the experimental group, C: 14th day of postnatal life in the experimental group, D: 21st day of postnatal in the experimental group. E: Birth in the control group, F: 7th day of postnatal life in the control group, G: 14th day of postnatal life in the control group, H: 21st day of postnatal in the control group.

The overall composition of infant oral flora at each time point was analyzed by PCoA analysis. The results show that the difference in beta diversity between the two groups at the 1st, 7th, 14th, and 28th days of life was not significant, suggesting that the oral flora structure of the two groups of infants was similar. however, we found that the microbiota composition was impacted by time (*p* = .001) (Figure 5).

**Figure 6:**
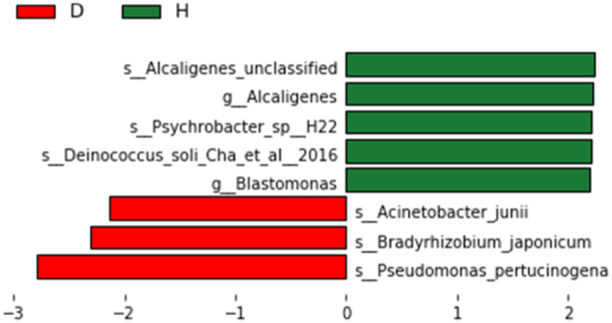
LEfSe analysis between the study and control groups at day of life 21 (LDA > 2). D: 21st day of postnatal in the experimental group. H: 21st day of postnatal in the control group.

Significant increases in the relative abundance of Alcaligenes and Blastomonas (at the genus level), as well as Deinococcus_soli_Cha_et_al_2016 and Psychrobacter_sp_H22 (at the species level), were observed in the control group in comparison with the study group. However, significant increases in the relative abundance of Acinetobacter_junii, Bradyrhizobium_japonicum, Pseudomonas_pertucinogena (at the species level) were observed in the study group (Figure 6).

**Table 3:** Proportions of Streptococcus, Staphylococcus, Acinetobacter, and Burkholderia‐Caballeronia‐Paraburkholderi (%).

Genus	A	B	C	D	E	F	G	H
*Streptococcus*	1.35	29.74	56.03	54.47	1.32	27.73	42.11	57.41
*Staphylococcus*	0.99	25.20	19.81	21.87	0.99	16.05	31.05	18.81
*Acinetobacter*	12.11	5.65	4.51	2.69	12.20	7.57	4.92	4.02
*Burkholderia‐Caballeronia‐Paraburkholderia*	8.77	4.50	1.90	1.55	9.36	5.89	2.03	1.45

*Note*: A: Birth in the experimental group, B: 7th day of postnatal life in the experimental group, C: 14th day of postnatal life in the experimental group, D: 21st day of postnatal in the experimental group. E: Birth in the control group, F: 7th day of postnatal life in the control group, G: 14th day of postnatal life in the control group, H: 21st day of postnatal in the control group.

When comparing the incidence of NEC, ventialtor‐associated pneumonia (VAP), and late‐onset sepsis (LOS) between the two groups, the differences were not statistically significant (Table 4).

## 4 DISCUSSION

Oral cavity is a complex ecosystem. At present, the early oral microbial colonization of very low birth weight infants is still controversial. One study has shown that the placental microbiota profiles were most similar to the infant oral microbiota.^15^ Oral microorganisms such as Fusobacterium nucleatum (a gram‐negative oral anaerobic) may facilitate blood‐borne transmission in the placenta.^16^ The diversity and composition of the oral microbiota is constantly changing after the birth of the foetus. At the phylum level, the oral microbiota of very low birth weight infants were dominated by Firmicutes, Proteobacteria, Actinobacteria, and Bacteroidetes, with the four dominant species accounting for more than 90% of the total macrobiota, which is similar to the proportion of dominant intestinal microbiota of preterm infants.^17^ The proportion of Firmicutes in the total oral phylum of tube‐fed very low birth weight infants increased progressively from the 1st postnatal day to the 21st day of life and peaked at the 21st day of life. This is similar to the microbial composition of the normal neonatal oral. In the anodontic phase, the abundance of the Firmicutes phylum in the oral microbiota is much higher than in other phyla.^18^ However, the abundance of Proteobacteria declined gradually and was the lowest at the 21st postnatal day. The abundance of Actinobacteria and Bacteroidetes decreased with increasing age. At the genus level, Streptococcus and Staphylococcus vastly dominated the oral microbiota of preterm infants, and the strong adhesion of Streptococcus to mucosal surfaces suggested that these two bacteria were early colonizing oral resident bacteria. During the first month of life, Streptococcus, Staphylococcus dominate the oral flora in normal newborns.^19^ Thus the early colonization of oral microbiota in tube‐fed very low birth weight infants is approximately the similar to that of normal newborns.

**Table 4:** Clinical outcomes between two groups at the time of discharge.

	Study group	Control group	*t/χ* ^2^	*p*
Necrotizing enterocolitis	2 (3.8)	3 (5.9)	0.276	.599
Ventialtor‐associated pneumonia	2 (3.8)	4 (7.8)	0.838	.360
Late‐onset sepsis	1 (1.9)	1 (2.0)	0.001	.978
Intraventricular hemorrhage	7 (13.7)	14 (27.4)	3.272	.070
Mortality	1 (1.9)	2 (4.0)	0.384	.535
Days to discharge	54.471 ± 17.389	59.941 ± 24.322	−1.323	.085

It is very important to explore the changes of oral microbiota of infants in the early stage of life for the health of the body and prevention of diseases. The diversity of the oral microbiota was highest on the first day of life and significantly dropped over time in infants across both groups. The structure of the oral microbiota of infant on the first and 7th day of life was similar. There was no significant change in the diversity of oral microbiota between the 14th and 21st postnatal day. The period from the 7th to the 14th day after birth was an important time for the development of the infant oral microbiota. Similar results were found in the experimental group. Even though preterm infants accepted oropharyngeal administration of mother's milk, there was no effect on the trend of oral microbiota in early life. It was consistent with the results of Romano‐Keeler et al.^20^ Oropharyngeal administration of mother's milk had neither an effect on overall oral bacterial diversity and composition. The bacterial diversity of newborns was highest at birth but rapidly declined within 30 days after birth. This may have been similar to intestinal flora community succession in preterm infants. Convergence of the infant gut microbiota towards decreasing beta‐diversity over time scales from months to years.^21^, ^22^ It may also have been associated with the use of antibiotics. Early empirical antibiotics have a sustained influence on the gut microbiota of preterm infants. Infants who received empiric antimicrobial agents in the first week had been lower bacterial diversity.^23^ Moreover, the presence of species belonging to Fusobacterium, Veillonella and Lactobacillus was also associated with antibiotics intake in the oral cavity of infants.^24^ The placenta and amniotic fluid are important sources of early oral microbiota for preterm infants.^25^ After birth, as the time of separation from the mother increases, the main factors influencing the diversity and richness of the oral microbiota change gradually from perinatal maternal factors such as contamination of amniotic fluid, gestational diabetes, oral caries, etc. to the environment, antibiotics, breast milk, etc.^26^, ^27^ Currently, most studies on oral flora are cross‐sectional study and have small sample sizes. There has been lack of large sample prospective cohort studies, especially in this particular population of preterm infants. By understanding the changes in the oral flora of preterm infants, we can better help them to establish the normal oral microbiological homeostasis and promote their growth and development.

The results of this study showed that there was no statistically significant difference between the Shannon Index and Simpon Index of the oral microbiota in the experimental and control groups within 21 days after birth, and the beta diversity was not statistically different. The results of Thatrimontrichai et al.^28^ were similar to this study. Therefore, we hypothesize that administration of oropharyngeal milk has no effect on the structure and diversity of the early oral microbiota in very low birth weight infants. This may have been attributed to the fact that the infants have been in the same environment since birth. The surfaces of the objects in the intensive care unit have common colonizing bacteria of the neonatal oral cavity, such as Pseudomonas, Streptococcus, Staphylococcus, Escherichia, etc.^29^ Previous study has found that environmental factors have a greater impact on oral microbiota than genetic factors.^30^ It has also been shown that the composition of the oral microbiota of infants who were delivered vaginally was more similar to the mother's vaginal microbiota than that of infants who were delivered by cesarean section.^31^ There was no statistical difference in the mode of delivery between the two groups in this study. The colonization of the early oral microbiota of preterm infants is susceptible to a variety of factors. These factors may therefore have contributed to the similar structure and diversity of the early oral microbiota between the two groups. However, Abd Elgawad M found significant reductions in Klebsiella species when the duration of administration of oropharyngeal milk was extended to full oral feeding. The number of days to reach complete oral feeding was 38.53 ± 5.5 days and 48.09 ± 8.1 days for the two groups, respectively.^32^ But, the last sampling time in this study was on the 21st day. Future studies could extend the intervention to explore the effect of administration of oropharyngeal milk on the oral microbiota of preterm infants.

Our research also found that significant increases in the relative abundance of Alcaligenes and Blastomonas (at the genus level), as well as Deinococcus_soli_Cha_et_al_2016 and Psychrobacter_sp_H22 (at the species level), were observed in the control group in comparison with the study group. However, Significant increases in the relative abundance of Acinetobacter_junii, Bradyrhizobium_japonicum, Pseudomonas_pertucinogenawere (at the species level) observed in the study group on the 21st day. Alcaligenes and Acinetobacter_junii are gram‐negative bacteria that may cause respiratory tract infections, septicaemia, endocarditis, and genitourinary tract infections. Usually, they survive in the host without causing any disease, and are susceptible to infection when the body's resistance is weakened.^33^, ^34^ Previous study has found that Planococcaceae is the dominant family in the colostrum group at 48 and 96 h.^14^ This may have been related to the different time of sample collection. In addition, large sample sizes, metagenomics and other methods are necessary to apply in subsequent experiments to better understand the effect of oropharyngeal Administration of mother's milk on infants’ oral microbes.

Previous research has hypothesized that oropharyngeal administration of mother's milk protect infants against NEC via several mechanisms: (1) cytokine interaction with oropharyngeal immune cells, (2) barrier protection against pathogens, (3) mucosal absorption of protective biofactors, among others.^12^ In our study, when comparing the incidence of NEC, VAP and LOS between the two groups, the differences were not statistically significant. Although the average number of hospital days was reduced by 5 days in the experimental group compared to the control group, the difference was not statistically significant. In comparison with the study by Romano‐Keeler et al.,^20^ the results were similar. But oral application of mother's own milk decreased LOS in preterm neonates in the study of Sudeep et al.^35^ Several studies have proven that this intervention is a feasible and safe for infants.^36^, ^37^ A 5‐year multicenter randomized controlled trial of 622 infants has been ongoing to explore the effect of this intervention on infectious complications such as NEC in preterm infants, with the expectation that it will provide a more positive theoretical basis for the clinical application.^38^

## 5 LIMITATIONS

This work is not without its limitations. First, We do not have environmental swabs from the NICU, which may contribute to the development of the oral microbiota. The research results may have limited generalizability as our study participants were recruited from a hospital. Nevertheless, our study examined the oral microbial colonization in very low birth weight infants during the first 21 days of life. Further research should explore the evolution of oral microbiota in infants during the transition from tube feeding to oral feeding, particularly the colonization of flora associated with potential infections.

## 6 CONCLUSION

This study shows that oropharyngeal mother's milk administration did not change the diversity and structural composition of the oral microbiota from birth to 21 days after birth. Furthermore, The oral microbial diversity of infants fed by gastric tube declined significantly over time. Firmicutes had replaced Proteobacteria as the predominant phylum.

## AUTHOR CONTRIBUTIONS

Jie Liu and Lilian Chen drafted the initial manuscript and participated in the analysis. Chuanzhong Yang, Xiaoyun Xiong, and Yan Ning carried out the analyses, reviewed and revised the manuscript. Jie Liu, Xiaohe Mu, and Xiyang Zhang, and Qian Zhao participated in data collection process. Lilian Chen and Xiaoling Qin designed the data collection instruments and coordinated and supervised data collection. All authors have read and agreed to the published version of the manuscript.

## CONFLICT OF INTEREST STATEMENT

The authors declare no conflicts of interest.

## ETHICS STATEMENT

The study was conducted according to the guidelines of the Declaration of Helsinki, and approved by the Ethics Committee of Shenzhen Maternity and Child Healthcare Hospital (SFYLS[2020]038). Informed consent was obtained from all subjects involved in the study.

## Data Availability

The data sets used and analyzed during the current study are available from the corresponding authors upon reasonable request. Sequencing data that support the findings of this study have been deposited in GSA and can be accessed with the BioProject ID PRJCA018129.
